# Preliminary marker-based validation of a novel biplane fluoroscopy system

**DOI:** 10.1186/1757-1146-5-S1-O36

**Published:** 2012-04-10

**Authors:** Joseph M Iaquinto, Richard Tsai, Michael Fassbind, David R Haynor, Bruce J Sangeorzan, William R Ledoux

**Affiliations:** 1 VA RR&D Center of Excellence for Limb Loss Prevention and Prosthetic Engineering, Seattle, WA, 98108, USA; 2 Departments of Radiology and Bioengineering, University of Washington, Seattle, WA, 98195-7117, USA; 3 Department of Orthopaedics & Sports Medicine, University of Washington, Seattle, WA, 98195-6500, USA; 4 Department of Mechanical Engineering, University of Washington, Seattle, WA, 98195, USA

## Background

The use of biplane fluoroscopy to track bones in the foot is challenging, due to distortion, overlap and image artefact inherent in fluoroscopy systems and high speed photography. The accuracy and precision of these systems have been reported [1-4] and are presented here for our biplane fluoroscopy system.

## Materials and methods

**Figure 1:**
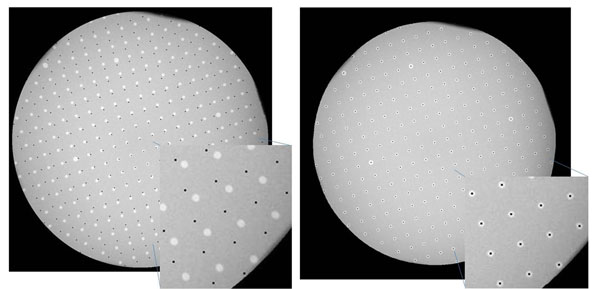
Distortion plate before (left) and after (right) correction for pin-hole distortion and magnetic distortion.

Biplane Fluoroscopy System: The system consists of two Philips BV Pulsera C-arms set in custom frames around a raised floor with a radiolucent imaging area. X-ray images are captured with high speed (1000fps) cameras. Validation Object: 1.6mm tantalum beads were placed in a machined block (wand) then measured to 7 microns with a Coordinate Measuring Machine to determine their centroid location. The wand was translated and rotated via a 1 micron precision stepper-motor for static validation, as well as manually swept through the field of view at ~0.5m/s for dynamic. Static Accuracy and Precision: accuracy was defined as the RMS error between the translation of the stepper-motor and the measured movement of the beads; precision is defined as the standard deviation of the bead locations. For rotation, accuracy was defined as the RMS error between the applied and measured rotation of the wand. Dynamic Accuracy and Precision: accuracy was defined as the RMS error between the known and measured inter-bead distance; precision was the standard deviation of the inter-bead distance. 3D location processing was accomplished using custom software written in MatLab to derive the 3D location of objects from two, time-synchronized, 2D fluoroscopy images of known spatial relationship. This software also compensates for the image distortion (Figure 1).

## Results

Translation: the overall RMS error was 0.066 mm, with a precision of ± 0.016 mm. Rotation: the RMS error was 0.125°. Dynamic motion: the overall RMS error was 0.126 mm, with a precision of ± 0.122 mm.

## Conclusions

The accuracies and precision in the results are comparable to similar such systems in development to investigate other joints of the body [1-4]. We are currently developing and validating a marker-less technique for tracking the bones of the foot.

## References

[B1] Brainerd EL X-ray reconstruction of moving morphology (XROMM): precision, accuracy and applications in comparative biomechanics research J Exp Zool A Ecol Genet Physiol 2010 313 262 279 20095029 10.1002/jez.589

[B2] Miranda D Accuracy and precision of 3-D skeletal motion capture technology56th ORS 2010 Paper no. 334

[B3] Li G Validation of a non-invasive fluoroscopic imaging technique for the measurement of dynamic knee joint motion J Biomech 2008 41 1616 1622 10.1016/j.jbiomech.2008.01.034 18394629

[B4] Kaptein BL A comparison of calibration methods for stereo fluoroscopic imaging systems J Biomech 2011 44 2511 2515 10.1016/j.jbiomech.2011.07.001 21783196

